# Editorial: Human Rights and Mental Health: Current Developments in Competence Assessment and Supported Decision-Making

**DOI:** 10.3389/fpsyt.2021.682606

**Published:** 2021-04-21

**Authors:** Matthé Scholten, Penelope June Weller, Scott Y. H. Kim, Jochen Vollmann

**Affiliations:** ^1^Institute for Medical Ethics and History of Medicine, Ruhr University Bochum, Bochum, Germany; ^2^Global and Social Studies Centre, RMIT University Melbourne, Melbourne, VIC, Australia; ^3^Department of Bioethics, National Institutes of Health, Clinical Center, Bethesda, MD, United States

**Keywords:** UN-CRPD, informed consent, mental capacity, competence to consent, supported decision-making, substitute decision-making, mental health care, psychiatry

This Research Topic presents the results of the international and interdisciplinary workshop Human Rights and Mental Health, held at the Institute for Medical Ethics and History of Medicine of the Ruhr University Bochum, Germany, on April 1–5, 2019. The workshop was organized by Matthé Scholten, Sarah Potthoff, Astrid Gieselmann, Jakov Gather, and Jochen Vollmann and funded by the German Federal Ministry of Education and Research. The workshop focused on ethical and legal issues surrounding the implementation of the United Nations (UN) Convention on the Rights of Persons with Disabilities (CRPD) in mental health care.

The CRPD was adopted by the UN General Assembly in 2006 and entered into force in 2008. One hundred and eighty-two states parties have ratified the convention to date. The convention articulates the rights laid down in general human rights documents for persons with disabilities, including persons with psycho-social disabilities or mental disorders. The CRPD has far-reaching implications for mental health care, and in particular for current practices of informed consent, capacity assessment, supported decision-making, substitute decision-making, and involuntary hospitalization and treatment. In particular the interpretation of CRPD article 12 by the Committee on the Rights of Persons with Disabilities sparked great controversy (1–6).

A unique feature of the workshop was that it brought together proponents of both radical CRPD and capacity-based models. The participants in the workshop were based in Germany, Ireland and the United Kingdom. Expert speakers included Peter Bartlett (Nottingham), Eilionóir Flynn (Galway), Jill Stavert (Edinburgh), and Martin Zinkler (Heidenheim), Margret Osterfeld (former Member of the UN Subcommittee on Prevention of Torture and other Cruel, Inhuman or Degrading Treatment or Punishment) and Theresia Degener (former Chairperson of the UN Committee on the Rights of Persons with Disabilities).

This Research Topic aims to enrich the sometimes heavily politicized debate on the CRPD with empirical data, legal arguments, ethical analyses, and clinical innovations to facilitate progress and reform at the level of policy and practice.

## Contributions To The Research Topic

The articles by Peter Bartlett, Jill Stavert, and Kevin De Sabbata explore the implications for the CRPD for mental health law and policy. Taking the Mental Capacity Act (2005) of England and Wales as an example, Bartlett argues that elements of domestic capacity-based laws can be successfully incorporated in CRPD-compliant systems. While challenges arise from the role of mental capacity as a gateway concept and the role of objective factors in determinations of best interest, Bartlett contends that these challenges are not insurmountable. Stavert emphasizes the positive obligation of states parties to the CRPD to work toward the equal enjoyment of human rights. In the context of mental health law reform in Scotland, she argues that legal mechanisms such as advance directives, enduring powers of attorney and mental health advocates can be developed to achieve closer alignment with the CRPD. Focusing on people with dementia, De Sabbata describes five key shortcomings of capacity-based models and argues that these limitations point to an urgent need for the implementation of decision-making frameworks based on the CRPD.

The articles first authored by McWilliams, Curk and Gurbai explore the current practice of capacity assessment in the United Kingdom. McWilliams et al. investigated the extent to which the results of neuroscientific and psychological measurements are used as evidence in determinations of mental capacity in England's Court of Protection. They found that no standardized measurements were used in most cases. Where standardized measurement was used, this mostly concerned the Mini Mental State Examination (MMSE), notwithstanding that MMSE scores cannot be used as a proxy for mental capacity (7). A standardized measure of mental capacity was used only in a single case.

Gurbai et al. scrutinized the use of the notion of “insight” in determinations of mental capacity before the Court of Protection. Although insight is not a criterion for mental capacity under the Mental Capacity Act (2005), Gurbai et al. found that the notion played an important role in a substantial share of capacity determinations before the court. There was not a single case in which insight was defined, the notion had a wide variety of meanings across cases and an alleged lack of insight was mostly not supported by evidence from structured instruments.

Curk et al. explore this notion further by means of a review and critique of standardized instruments to assess insight. Where insight can be defined neutrally as an awareness of one's own health condition and an awareness of the possibilities of treatment, Curk et al. argue that most instruments operationalize insight as agreement with the diagnosis and compliance with recommended treatment. They then proceed to argue that this operationalization of insight is paternalistic and ethically problematic.

The article by Stephenson et al. systematizes the available empirical evidence on a promising clinical intervention to promote the autonomy of mental health service users: so-called psychiatric advance directives (PADs). The specific focus of the systematic review is on PADs in bipolar disorder. Stephenson et al. found that there is a high interest among people with bipolar in completing PADs in collaboration with a mental health professional to state both treatment requests and treatment refusals. Barriers to PAD completion included lack of awareness and lack of support, and benefits of PADs included avoidance of harm, faster recovery and a reduction of coercion.

All in all, the articles published in this Research Topic argue that current capacity and mental health laws as well as current practices of supported decision-making and capacity assessment should be improved. We hope that the novel legal mechanisms and clinical interventions proposed in the contributions to this Research Topic pave the way for improvements in policy and practice.

## Author Contributions

MS summarized the key findings of the empirical and conceptual articles and drafted the full article. PJW summarized the key findings of the legal articles. PJW, SYHK, and JV revised the drafts critically for important intellectual content. All authors made substantial contributions to the conception and design of the article, agreed with the article's content, and gave approval for the final version to be published.

## Conflict of Interest

The authors declare that the research was conducted in the absence of any commercial or financial relationships that could be construed as a potential conflict of interest.
